# Removal of a migrated biliary stent buried in a common bile duct stone using electrohydraulic lithotripsy

**DOI:** 10.1093/jcag/gwad062

**Published:** 2024-01-10

**Authors:** Tsuyoshi Suda, Masahiro Yanagi, Naoki Oishi, Eiki Matsushita

**Affiliations:** Department of Gastroenterology, Kanazawa City Hospital, 3-7-3, Heiwamachi, Kanazawa, Ishikawa 921-8105, Japan; Department of Gastroenterology, Kanazawa City Hospital, 3-7-3, Heiwamachi, Kanazawa, Ishikawa 921-8105, Japan; Department of Gastroenterology, Kanazawa City Hospital, 3-7-3, Heiwamachi, Kanazawa, Ishikawa 921-8105, Japan; Department of Gastroenterology, Kanazawa City Hospital, 3-7-3, Heiwamachi, Kanazawa, Ishikawa 921-8105, Japan

**Keywords:** common bile duct stone, electrohydraulic lithotripsy, migration, peroral cholangioscopy

An 84-year-old man was admitted for intrahepatic bile duct dilatation investigation. Magnetic resonance cholangiopancreatography and endoscopic retrograde cholangiography revealed a common bile duct (CBD) stone >2 cm in diameter (Figures 1A and 1B). Subsequently, a plastic stent (PS) was implanted (Figure 1C) and an electrohydraulic lithotripsy (EHL) procedure was scheduled. On follow-up, the PS was observed to have migrated proximally (Figure 1D) and could not be retrieved. Although the migrated PS was seen adjacent to the stone on fluoroscopic images, peroral cholangioscopy (POCS) revealed that the PS was embedded in the CBD stone (Figure 2A). Baskets and mechanical lithotripters failed to retrieve the buried PS or remove the stone. Consequently, EHL was conducted to enable stent removal. EHL crushed the CBD stone (Figure 2B), exposing the lower end of the PS (Figure 2C) and enabling retrieval of the PS through a snare (Figure 2D).

**Figure 1.**
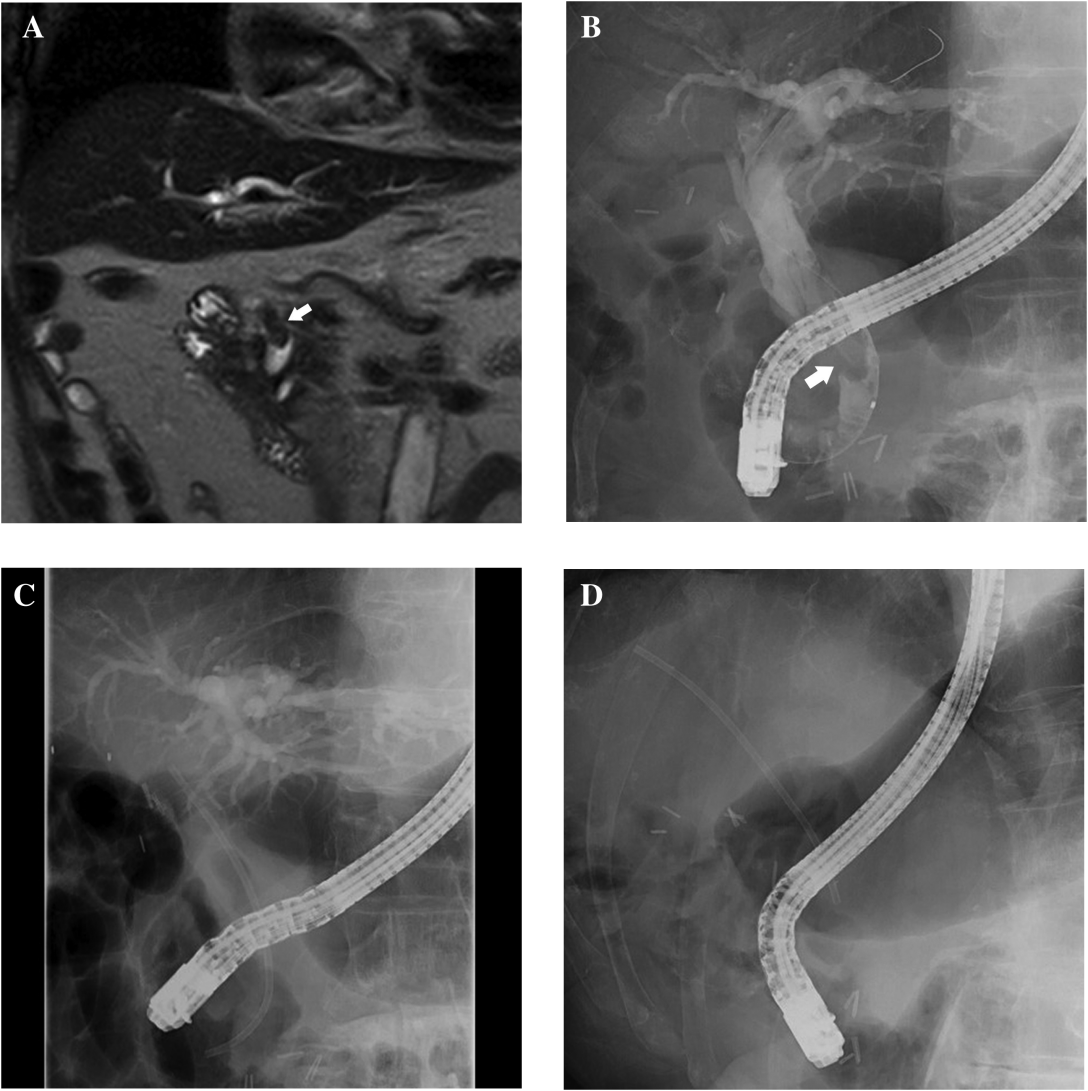
(A) and (B) A large common bile duct stone (arrow) seen on magnetic resonance cholangiopancreatography and endoscopic retrograde cholangiography. (C) Initial position of the plastic stent. (D) The plastic stent after migration.

**Figure 2.**
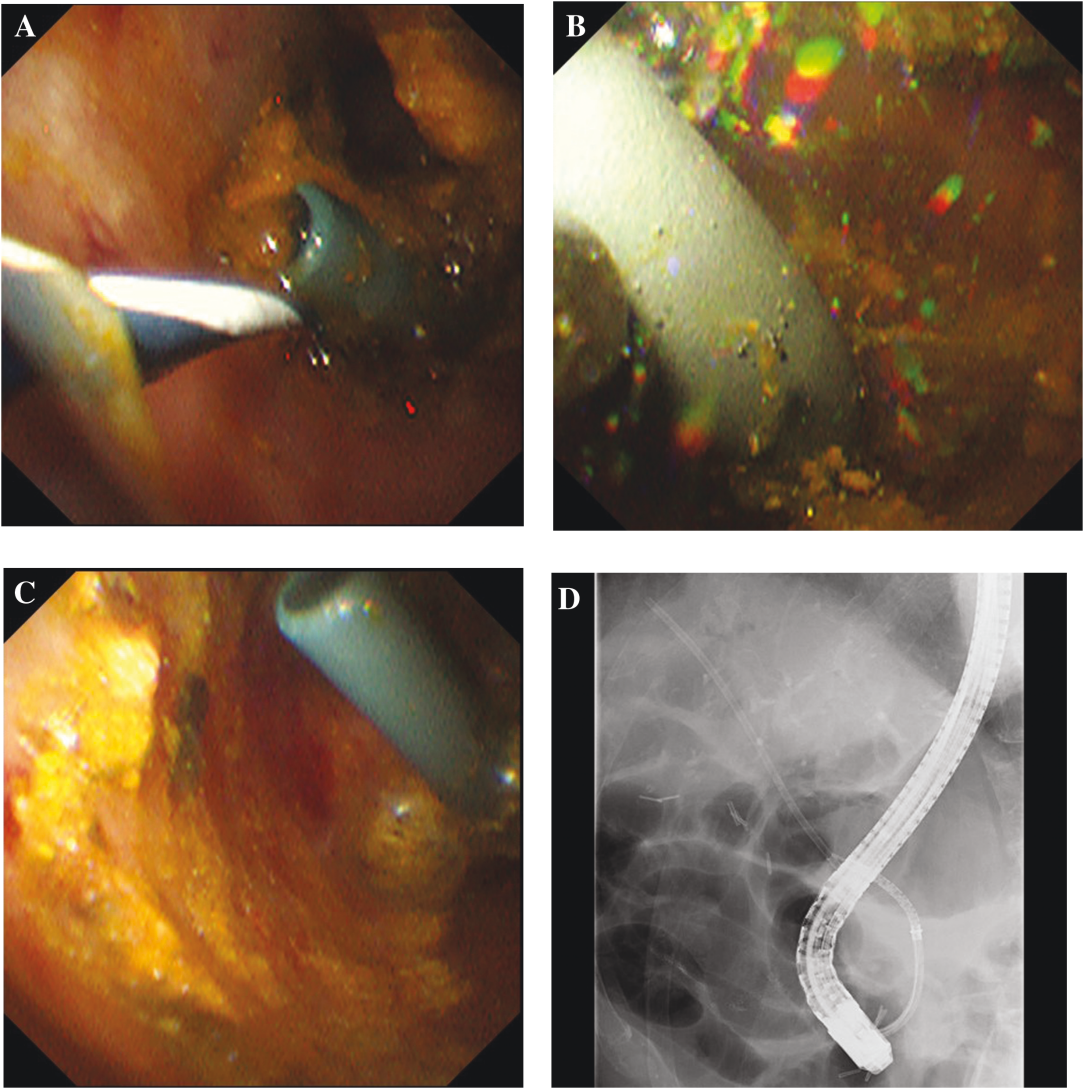
(A) On peroral cholangioscopy, the migrated plastic stent was seen lodged in the stone. (B) Implementation of electrohydraulic lithotripsy. (C) Following electrohydraulic lithotripsy, the lower end of the stent buried in the stone was exposed. (D) Successful grasping of the migrated plastic stent using a snare.

Biliary stent migration occurs in 5%–10% of patients as a complication of biliary drainage. Although both proximal and distal migration can occur, extraction of a proximally migrated stent is more problematic. Generally, the stent is removed using fluoroscopy with forceps, baskets, or balloons. Evidence from recent studies has established the viability of POCS in facilitating migrating stent removal.^1^ In this patient, the identification of the PS concealed inside the CBD stone was facilitated through POCS. Furthermore, EHL proved useful for extracting the PS buried in the CBD stone.

## Supplementary Material

gwad062_suppl_Supplementary_Materials

## Data Availability

The data that support the findings of this study are available from the corresponding author, TS, upon reasonable request.
